# COVID-19 knowledge, beliefs, prevention behaviours and misinformation in the context of an adapted seasonal malaria chemoprevention campaign in six northern Nigerian States

**DOI:** 10.1186/s41182-020-00288-7

**Published:** 2020-12-14

**Authors:** Sol Richardson, Taiwo Ibinaiye, Jamilu Nikau, Olusola Oresanya, Madeleine Marasciulo, Arantxa Roca-Feltrer, Christian Rassi, Olatunde Adesoro

**Affiliations:** 1 Malaria Consortium, The Green House, 244–254 Cambridge Heath Road, London, E2 9DA UK; 2 Malaria Consortium Nigeria, 33 Pope John Paul Street, Maitama, Abuja-FCT, Nigeria; 3 Nigerian National Malaria Elimination Programme, Orji Uzor Kalu House, Central Business District, Abuja-FCT, Nigeria; 4 Malaria Consortium USA, 8024 Upper Lake Drive, Raleigh, NC 27615 USA

**Keywords:** COVID-19, Nigeria, Seasonal malaria chemoprevention, Public health campaigns, Community health workers, Infodemics

## Abstract

**Background:**

Seasonal malaria chemoprevention (SMC) using sulfadoxine-pyrimethamine and amodiaquine is an efficacious intervention for protection of children against *Plasmodium falciparum* malaria during the rainy season. In response to the global COVID-19 pandemic, Malaria Consortium adapted its SMC delivery model to ensure safety of distributors, data collectors and beneficiaries. We conducted a SMC monitoring survey in July 2020 in the states of Bauchi, Jigawa, Kano, Katsina, Sokoto and Yobe, with questions on COVID-19 prevention behaviours and symptoms, and belief in misinformation. We investigated the associations between receipt of information on COVID-19 by different sources, including from SMC distributors, and these three outcomes using logistic generalised estimating equations. We also considered moderation of effectiveness of message delivery by SMC distributors and adherence to use of face coverings.

**Results:**

We obtained a representative sample of 40,157 caregivers of eligible children aged 3–59 months, of which 36,914 (91.92%) reported knowledge of COVID-19. The weighted proportions of respondents who correctly identified COVID-19 prevention behaviours and symptoms, and who reported belief in COVID-19 misinformation, were 80.52% (95% confidence interval [95% CI] 80.02–81.00), 81.72% (95% CI 81.23–82.20) and 22.90% (95% CI 22.24–23.57). Receipt of information on COVID-19 from SMC distributors during the campaign was significantly associated with higher odds of caregiver knowledge of COVID-19 prevention behaviours (odds ratio [OR] 1.78, 95% CI 1.64–1.94, *p* < 0.001) and symptoms (OR 1.74, 95% CI 1.59–1.90, *p* < 0.001) and lower odds of belief in COVID-19 misinformation (OR 0.92, 95% CI 0.85–1.00, *p* = 0.038). The associations between message delivery by SMC distributors and the three outcomes were moderated by their adherence to face covering use. Receipt of information by other sources used to deliver government public health messages, including radio and health facility workers, was also associated with knowledge of COVID-19.

**Conclusions:**

Malaria Consortium’s SMC programme was successfully adapted in the context of COVID-19 and was a conduit for high-quality public health messages. Standard SMC monitoring and evaluation activities can be adapted to gather evidence on emerging public health issues such as the global COVID-19 pandemic.

## Background

Seasonal malaria chemoprevention (SMC) using one dose of sulfadoxine-pyrimethamine (SP) in combination with three daily doses amodiaquine (AQ) has been shown to be an efficacious [1–4] and cost-effective [5] intervention for prevention of *Plasmodium falciparum* malaria. The World Health Organization (WHO) recommends SMC administration to children aged 3–59 months in areas of high transmission to provide chemotherapeutic protection during the rainy season, when most deaths occur [1]. In 2020, Malaria Consortium supported SMC campaigns in Nigeria, Burkina Faso and Chad, covering a target population of 12.39 million eligible children. In Nigeria, SMC is delivered door-to-door over four consecutive monthly cycles spanning July to October by trained voluntary SMC community distributors [6].

In response to the ongoing global COVID-19 pandemic, the global SMC community in collaboration with Malaria Consortium published guidelines [7] to support adaptation of SMC delivery to ensure safety of SMC distributors, programme beneficiaries and communities. Malaria Consortium created a job aid [8] and a training flipbook to guide the safe administration of SMC by community distributors^1^. These adaptations also include dissemination of health messages^2^ about prevention of COVID-19 to all caregivers of eligible children [8].

While there exists some evidence on knowledge and attitudes of COVID-19 in various settings, including Nigeria [9–13], there remains, to our knowledge, no evidence on effectiveness of different sources of information that influence these outcomes. Diffusion of misinformation on COVID-19 has emerged as a significant issue worldwide [14], including in Nigeria, where ‘myths’ surrounding methods to prevent or cure infection have the potential to undermine public health messaging and interventions [15].

The purpose of this study was to describe the prevalence of knowledge of COVID-19 prevention behaviours and common symptoms suggestive of COVID-19, and belief in misinformation about COVID-19 among caregivers of children eligible for SMC in six Nigerian states covered by the SMC programme, and to investigate the relationship between these outcomes and receipt of COVID-19 information by SMC distributors and other 5were collected from 18 July to 4 August 2020 as part of the routine cross-sectional end-of-cycle survey for SMC cycle 1 in the states of Bauchi, Jigawa, Kano, Katsina, Sokoto and Yobe^3^. Time between completion of the SMC cycle and the start of surveys ranged from 6 days (Sokoto) to 9 days (Bauchi and Kano). The survey employed the lot quality assurance sampling (LQAS) methodology [16] to obtain data on SMC coverage and other related indicators to assess quality of programme delivery at the ward level, which provides a sample of respondents equivalent to that from a cluster randomised survey when data are pooled across multiple sampling units^4^. The questionnaire form was written in English and interpreted into Hausa (the regional lingua franca) by data collectors during household visits in all states. Respondents were female caregivers of children aged 3–59 months randomly sampled within residential compounds containing at least one eligible child^5^.

Additional questions related to COVID-19 were included in the survey for the purpose of this study, which respondents were asked if they reported that they had ever heard of ‘COVID-19’ or ‘coronavirus disease’ (or local variants).

Three outcomes were investigated. Knowledge of COVID-19 prevention was assessed based on spontaneous identification of at least one COVID-19 prevention behaviour listed on Malaria Consortium’s SMC ‘job aid’ [8]^6^. Knowledge of symptoms was assessed based on spontaneous identification of at least one COVID-19 symptoms listed on the United States Centers for Disease Control and Prevention (CDC) website [17]^7^. Belief in COVID-19 misinformation was defined as an incorrect answer to at least one of three ‘true or false’ questions on three common COVID-19 ‘myths’ mentioned on the Nigeria Centre for Disease Control (NCDC) website [18]^8^.

Respondents in compounds visited by SMC distributors were asked whether they had received information on COVID-19 from them and whether distributors were wearing masks or face coverings^9^ during their visit. Respondents aware of COVID-19 were queried on other sources from which they received information on COVID-19^10^.

After undertaking a descriptive analysis of each variable by state, we employed logistic generalised estimating equations with an exchangeable correlation structure to account for clustering of responses within wards to investigate the associations between information sources and the three outcomes [19]. Post-sampling weights based on ward and state population sizes were applied. Three models were fitted: model 1 tested the univariate association between receipt of information on COVID-19 from SMC distributors and each outcome; model 2 categorised receipt of information according to whether distributors wore face coverings; model 3 further adjusted for receipt of information on COVID-19 from other sources.

## Results

Of a total 40,157 respondents, 36,914 (91.92%^11^) reported awareness of COVID-19. The weighted proportions of respondents who correctly identified COVID-19 prevention behaviours and symptoms, and who reported belief in COVID-19 misinformation, were 80.52% (95% confidence interval [95% CI] 80.02–81.00), 81.72% (95% CI 81.23–82.20) and 22.90% (95% CI 22.24–23.57) respectively across the six states investigated (Table 1). Across all states, 50.61% (95% CI 49.87–51.34) received information on COVID-19 from SMC distributors, compared with 70.75% (95% CI 70.14–71.35) by radio and 46.67% (95% CI 45.94–47.39) by word of mouth. The results show wide differences in outcomes and information sources across states.

**Table 1:** Summary statistics for awareness of COVID-19, correct identification of COVID-19 prevention behaviours and COVID-19 symptoms, belief in COVID-19 misinformation, provision of information on COVID-19 by SMC community drug distributors and other sources of information on COVID-19 in the states of Bauchi, Jigawa, Kano, Katsina, Sokoto and Yobe, Nigeria (*n* = 40,157)

Variable (COVID-19 awareness and knowledge outcomes)		Bauchi*	Jigawa*	Kano*	Katsina*	Sokoto*	Yobe*	Six states**
		% (95% CI)	% (95% CI)	% (95% CI)	% (95% CI)	% (95% CI)	% (95% CI)	% (95% CI)
Aware of COVID-19^†^		80.32 (78.94–81.62)	97.17 (96.67–97.61)	95.12 (94.39–95.76)	90.11 (89.41–90.77)	94.31 (93.65–94.90)	89.56 (88.35–90.65)	93.00 (92.64–93.38)
Knowledge of COVID-19 prevention^‡^		47.07 (45.02–49.13)	85.20 (84.23–86.11)	86.88 (86.12–87.60)	70.37 (69.17–71.54)	80.81 (79.51–82.03)	86.48 (85.15–87.71)	80.52 (80.02–81.00)
Knowledge of symptoms suggestive of COVID-19^‡^		50.67 (48.60–52.75)	85.98 (85.03–86.88)	87.91 (87.14–88.64)	71.82 (70.64–72.97)	82.99 (81.79–84.13)	85.38 (84.01–86.65)	81.72 (81.23–82.20)
Belief in COVID-19 misinformation^‡^		34.24 (32.20–36.33)	24.10 (22.85–25.39)	25.20 (23.90–26.54)	23.44 (22.36–24.56)	8.87 (8.00–9.83)	22.35 (20.54–24.28)	22.90 (22.24–23.57)
Variable (sources on information on COVID-19)								
SMC distributor during campaign^‡^		18.13 (16.66–19.69)	63.49 (62.12–64.84)	51.36 (49.95–52.76)	32.99 (31.80–34.20)	62.39 (60.70–64.05)	77.79 (76.01–79.47)	50.61 (49.87–51.34)
SMC distributor during campaign	Without face covering^‡^	1.13 (0.80–1.60)	1.45 (1.15–1.81)	2.98 (2.61–3.39)	3.37 (2.92–3.88)	1.46 (1.16–1.84)	2.06 (1.63–2.60)	2.52 (2.33–2.74)
	Wearing face covering^‡^	17.00 (15.57–18.53)	62.04 (60.66–63.41)	48.38 (46.99–49.78)	29.62 (28.47–30.79)	60.93 (59.23–62.60)	75.73 (73.92–77.46)	48.08 (47.35–48.81)
Other information sources (non-exclusive categories)	Local leader^‡^	8.37 (7.28–9.60)	19.05 (17.95–20.21)	20.73 (19.53–21.99)	7.82 (7.17–8.51)	17.62 (16.42–18.90)	36.94 (34.74–39.20)	17.87 (17.29–18.51)
	Religious leader^‡^	7.39 (6.32–8.61)	11.40 (10.47–12.39)	12.77 (11.68–13.94)	6.21 (5.63–6.85)	9.04 (8.12–10.06)	30.96 (28.88–33.11)	11.69 (11.16–12.26)
	Health facility staff^‡^	5.72 (4.80–6.81)	16.21 (15.10–17.37)	14.23 (13.27–15.25)	11.66 (10.89–12.48)	22.58 (20.84–24.41)	41.52 (39.45–43.61)	16.39 (15.85–16.95)
	CHW^‡^	4.48 (3.74–5.35)	20.99 (19.79–22.25)	11.67 (10.79–12.61)	10.39 (9.68–11.15)	23.92 (22.54–25.37)	35.89 (33.96–37.86)	15.41 (14.91–15.91)
	Radio^‡^	46.38 (44.32–48.44)	70.61 (69.36–71.83)	81.89 (80.89–82.84)	58.32 (57.03–59.61)	69.19 (67.63–70.71)	60.27 (58.12–62.38)	70.75 (70.14–71.35)
	Printed materials^‡^	0.11 (0.01–0.32)	1.09 (0.79–1.50)	3.10 (2.52–3.80)	1.73 (1.44–2.08)	2.33 (1.87–2.89)	6.47 (5.47–7.64)	2.53 (2.25–2.85)
	Television^‡^	4.03 (3.29–4.93)	7.02 (6.29–7.82)	16.56 (15.29–17.91)	11.29 (10.34–12.31)	10.09 (8.99–11.31)	24.55 (22.63–26.58)	13.34 (12.71–14.00)
	Town announcer^‡^	10.15 (8.57–74.85)	14.71 (13.72–15.75)	8.90 (8.14–9.73)	14.38 (13.52–15.28)	20.03 (13.29–16.15)	14.66 (13.29–16.15)	12.64 (12.19–13.11)
	Word of mouth^‡^	72.96 (70.97–74.85)	56.38 (54.96–57.79)	37.20 (35.90–38.53)	50.02 (48.69–51.35)	56.47 (54.74–58.19)	39.97 (37.90–42.07)	46.67 (45.94–47.39)
	Any other source^‡^	5.40 (4.65–6.26)	11.21 (10.35–12.14)	1.87 (1.45–2.40)	3.24 (2.83–3.70)	2.04 (1.74–2.47)	2.41 (1.93–3.01)	3.36 (3.34–3.38)

*Abbreviations*: *CHW* community health worker

*Results adjusted using post-sampling weights based on ward population size

**Weighted average for the population surveyed across the six states investigated, adjusted using post-sampling weights based on ward and state population sizes (total state populations, or in the case of Bauchi, the combined population of the local government areas covered by the SMC programme and LQAS survey (Dambam, Darazo, Gamawa, Giada, Itas Gadau, Jamaare, Katagum, Misau, Shira and Zaki)

^†^Sample sizes: Bauchi (*n* = 4,115), Jigawa (*n* = 6,940) Kano (*n* = 11,393) Katsina (*n* = 8,626) Sokoto (*n* = 5,567), Yobe (*n* = 3,706), and six-state total (*n* = 36,914)

^‡^Sample sizes (restricted to respondents who reported awareness of COVID-19): Bauchi (*n* = 3,248), Jigawa (*n* = 6,771), Kano (10,904), Katsina (7,448), Sokoto (*n* = 5,115), Yobe (*n* = 3,348), and six-state total (*n* = 40,157)

The statistical analysis found that receipt of information on COVID-19 from SMC distributors during the campaign was significantly associated with around 75% higher odds of caregiver knowledge of COVID-19 prevention behaviours and symptoms and negatively associated with belief in COVID-19 misinformation (Table 2, model 1).

**Table 2:** Results of generalised estimating equations for associations between sources of information on COVID-19 and correct identification of COVID-19 prevention behaviours, COVID-19 symptoms, and belief in COVID-19 misinformation in the states of Bauchi, Jigawa, Kano, Katsina, Sokoto and Yobe, Nigeria (*n* = 36,914)

Model	Variable (sources on information on COVID-19)		Knowledge of COVID-19 prevention behaviours		Knowledge of symptoms suggestive of COVID-19^‡^		Belief in COVID-19 misinformation	
			OR (95% CI)	*p*	OR (95% CI)	*p*	OR (95% CI)	*p*
Model 1*	SMC distributor during campaign		1.78 (1.64–1.94)	< 0.001	1.74 (1.59–1.90)	< 0.001	0.92 (0.85–1.00)	0.038
Model 2*	SMC distributor during campaign	Without face covering	1.35 (1.13–1.61)	0.001	1.44 (1.23–1.69)	< 0.001	1.11 (0.91–1.35)	0.320
		Wearing face covering	1.84 (1.69–2.01)	< 0.001	1.78 (1.62–1.95)	< 0.001	0.90 (0.83–0.98)	0.012
Model 3*	SMC distributor during campaign	Without face covering	1.36 (1.12–1.67)	0.002	1.48 (1.22–1.80)	< 0.001	1.11 (0.91–1.35)	0.294
		Wearing face covering	1.83 (1.67–2.01)	< 0.001	1.75 (1.57–1.94)	< 0.001	0.92 (0.84–1.00)	0.042
	Other information sources (non-exclusive categories)	Local leader	1.20 (1.07–1.35)	0.002	1.35 (1.20–1.52)	< 0.001	0.96 (0.85–1.10)	0.574
		Religious leader	1.12 (0.97–1.29)	0.119	1.22 (1.02–1.46)	0.031	0.87 (0.72–1.07)	0.184
		Health facility staff	1.49 (1.32–1.69)	< 0.001	1.68 (1.47–1.92)	< 0.001	0.83 (0.71–0.97)	0.021
		CHW	1.35 (1.20–1.52)	< 0.001	1.67 (1.46–1.90)	< 0.001	0.99 (0.89–1.09)	0.790
		Radio	1.82 (1.65–2.00)	< 0.001	2.05 (1.84–2.28)	< 0.001	0.88 (0.81–0.95)	0.002
		Printed materials	0.92 (0.67–1.26)	0.607	0.93 (0.64–1.35)	0.695	0.72 (0.41–1.25)	0.244
		Television	1.71 (1.48–1.97)	< 0.001	1.78 (1.52–2.10)	< 0.001	0.90 (0.77–1.03)	0.132
		Town announcer	1.37 (1.22–1.53)	< 0.001	1.48 (1.29–1.70)	< 0.001	0.95 (0.84–1.08)	0.455
		Word of mouth	0.90 (0.83–0.98)	0.012	0.92 (0.84–1.01)	0.079	0.96 (0.86–1.08)	0.541
		Any other source	1.15 (0.97–1.37)	0.109	1.40 (1.05–1.86)	0.009	0.99 (0.84–1.17)	0.895

*Abbreviations*: *CHW* community health worker

*Results of generalised estimating equations for dichotomous outcomes (accounting for within-ward correlation of responses) using a logit link function and exchangeable correlation structure, expressed as odds ratios with 95% confidence intervals. Odds ratios show odds of each outcome among respondents receiving information from each information source, compared with those not receiving information from that source. The analytic sample was restricted to respondents who reported awareness of COVID-19. Post-sampling weights based on ward and state population sizes were applied

Similar associations were found for models 2 and 3, before and after adjustment for receipt of COVID-19 information through other sources. Receipt of information from SMC distributors not wearing face coverings was significantly associated with increased knowledge of COVID-19 prevention behaviours and symptoms; no association was found for belief in misinformation. Meanwhile, receipt of information from SMC distributors wearing face coverings was positively associated with knowledge of prevention behaviours (odds ratio [OR] 1.83, 95% CI 1.67–2.01, <0.001) and symptoms (OR 1.75, 95% CI 1.57–1.94, *p* < 0.001) with larger effect sizes and negatively associated with belief in misinformation (OR 0.92, 95% CI 0.84–1.00, *p* = 0.042) (model 3). Receipt of information via local leaders, health facility workers, CHWs, radio, television and town announcers was associated with knowledge of prevention behaviours. In addition to these sources, provision of information through religious leaders and other sources were positively associated with knowledge of symptoms. Word of mouth, after mutual adjustment for receipt of COVID-19 information via other sources including SMC distributors, was negatively associated with knowledge of COVID-19 prevention behaviours. Receipt of information via radio and health facility workers was negatively associated with belief in COVID-19 misinformation.

## Discussion

Dissemination of information on COVID-19 forms a critical part of prevention efforts; one study in north-central Nigeria found knowledge of causes, prevention behaviours and symptoms of COVID-19 were associated with more positive attitudes towards prevention measures implemented by authorities among respondents [20].

Differences in each of the three outcomes by state are likely driven, at least partially, by access to information on COVID-19, as evidenced by disparities in self-reported sources of information among respondents. While delivery of COVID-19 messages by SMC distributors was associated with all outcomes investigated, these associations were moderated by adherence of SMC distributors to wearing of face coverings. This is consistent with observations that effectiveness of health messaging is influenced by perceptions of credibility of health promoters, including their perceived adherence to messages delivered [21].

In Nigeria, the Federal Ministry of Health, in collaboration with agencies such as NCDC and the National Primary Health Care Development Agency, has undertaken a programme to disseminate messages on COVID-19 prevention and symptoms through health facility workers, with training provided by the federal government. Cascade training with standardised messages has similarly been centrally provided to local traditional leaders and religious leaders on public health messaging, identification of potential cases, case reporting to authorities, and measures to ensure safety of constituents and congregations [15]. NCDC public health information campaigns have been transmitted via radio and television, which have explicitly attempted to counter COVID-19 misinformation. Radio campaigns are of particular importance as the most common source of information. Word of mouth, independent of other information sources, may have spread rumours and misinformation on prevention behaviours, however [22].

Although the LQAS survey obtained a large, representative sample of caregivers of children eligible for SMC, the degree to which its findings can be generalised to the wider population (e.g. men and older people) is uncertain. Given its objective of rapidly assessing SMC programme coverage and quality of delivery over a wide geographic area, it was impractical to include questionnaire items on demographic variables to investigate individual-level predictors of the three outcomes. Reporting of information sources relied on caregiver recall. Another limitation was that of the language of the survey form; inconsistencies may have arisen in question phrasing during interpretation into Hausa, and difficulties in comprehension by non-native Hausa users may have been a cause of misreporting by caregivers. Responses indicating receipt of COVID-19 information via ‘word of mouth’ may have been a result of misinterpretation by both caregivers and data collectors. This category may have covered sources other than ‘family or friends’; interpretation of associations between receipt of information via word of mouth and study outcomes should therefore be interpreted with caution.

## Conclusions

While the results imply that national public health information campaigns in Nigeria were effective at increasing knowledge of COVID-19 and reducing belief in misinformation, vaccination or mass drug administration programmes, such as SMC, can also serve as conduits for high-quality public health messages and may complement efforts to reduce disease transmission. Their routine monitoring and evaluation activities, such as beneficiary surveys, may be quickly adapted to gather evidence on emerging public health issues such as the global COVID-19 pandemic.

## Data Availability

Original data and derived variables used in this study, and Stata syntax, are available from the corresponding author upon request.

## Abbreviations

95% CI: 95% confidence interval
AQ: Amodiaquine
CDC: United States Centers for Disease Control and Prevention
CHW: Community health worker
COVID-19: Coronavirus disease (2019)
LQAS: Lot quality assurance sampling
NCDC: Nigeria Centre for Disease Control
OR: Odds ratio
SMC: Seasonal malaria chemoprevention
SP: Sulfadoxine-pyrimethamine
WHO: World Health Organization

## Footnotes

1 Adaptations to standard SMC delivery included daily assessment of SMC distributors for potential COVID-19 caseness by temperature testing, use of alcohol-based hand sanitiser and masks or face coverings, two metre distancing between distributors and beneficiaries, triage of children and caregivers for potential COVID-19 caseness, reporting of possible cases of COVID-19 according to local guidelines, dissemination of health messages about prevention of COVID-19 to caregivers, disinfection of materials used in SMC delivery, safe disposal of materials using a bio-waste bag, and washing of hands and clothes after completion of SMC distribution.

2 Health messages communicated to caregivers included frequently washing hands with soap and water or alcohol-based hand sanitizer for 30 s; avoidance of touching eyes, nose or mouth with unclean hands or hands that have touched unclean surfaces; covering the nose and mouth with the inside of the elbow when sneezing or coughing; avoiding physical greetings and contact with others outside the household; refraining from attending public gatherings or events; avoiding spitting in public; distancing from people outside the household by two metres; staying at home as much as possible; wearing a face mask or face covering if sick or experiencing fever, cough, body aches or difficulty breathing; and wearing a face mask or face covering if caring for a person with COVID-19.

3 The SMC programme and cycle 1 LQAS survey provided coverage of all LGAs in targeted states, with the exception of Bauchi, where only Dambam, Darazo, Gamawa, Giada, Itas Gadau, Jamaare, Katagum, Misau, Shira and Zaki LGAs (representing the northern half of the state) were covered. The target population of eligible children aged 3–59 months in these areas totalled 8.65 million children. SMC cycle 1 took place over the following dates in each state: Bauchi (9–12 July), Jigawa (9–12 July), Kano (13–16 July), Katsina (9–12 July), Sokoto (9–12 July), Yobe (7–12 July). Data collection for the cycle 1 end-of-cycle survey took place over the following dates in each state: Bauchi (18–25 July), Jigawa (19–25 July), Kano (25 July to 4 August), Katsina (20–26 July), Sokoto (18–28 July), Yobe (20–29 July).

4 The LQAS survey was conducted with a lot size of 25 compounds per ward, so as to give a sufficient power to detect failure with α and β errors of < 0.1 based on a decision criterion of 70% and target of 90%. This multi-objective LQAS survey collected data on 17 key indicators, covering coverage of SMC, caregiver adherence to the treatment schedule, caregiver awareness and knowledge of SMC, and provision of information on COVID-19 and mask use by SMC distributors. Data were collected via the mobile application SurveyCTO (https://www.surveycto.com/).

5 A roster of all children aged 0–9 years within each compound was entered and one child aged 3–59 months was automatically selected at random by the mobile application. All questions regarding COVID-19 were posed to the primary caregiver of this child.

6 Respondents were requested to name as many prevention behaviours as possible. Spontaneous identification of one or more of the following, listed in Section 10 of the Malaria Consortium SMC job aid, was considered to indicate knowledge of COVID-19 prevention behaviours among caregivers: frequently wash your hands with soap and water or alcohol-based hand sanitiser for 30 s; do not touch your eyes, nose or mouth with unclean hands or hands that have touched unclean surfaces; cover your mouth and nose with the inside of your elbow when sneezing or coughing; avoid physical greetings and contact with others outside the household; do not attend public gatherings or events; avoid spitting in public; distance yourself from people outside the household by 2 m; stay home as much as possible; wear a face mask or face covering if you are sick or have fever, cough, body aches or difficulty breathing; wear a face mask or face covering if you are caring for a person with COVID-19. Caregivers were considered not to have knowledge of COVID-19 prevention behaviours if they could not provide a response, or only mentioned other prevention methods not listed.

7 Respondents were requested to list as many COVID-19 symptoms as possible. Spontaneous identification of one or more of the following, listed on the listed on the CDC website (https://www.cdc.gov/coronavirus/2019-ncov/symptoms-testing/symptoms.html), was considered to indicate knowledge of COVID-19 symptoms among caregivers: fever or chills; cough; shortness of breath or difficulty breathing; fatigue; muscle or body aches; headache; new loss of taste or smell; sore throat; congestion or runny nose; nausea or vomiting; diarrhoea. Caregivers were considered not to have knowledge of COVID-19 symptoms if they could not provide a response, or only mentioned other symptoms not listed.

8 These three ‘myths’ included: “taking antibiotics or antimalarial (such as chloroquine) can offer protection against being infected with COVID-19”; “chewing raw garlic can offer protection against being infected with COVID-19”; and “gargling salty water can offer protection against being infected with COVID-19”. Respondents were requested to answer “true” or “false” to each statement. After responding to all three questions, data collectors were instructed to inform respondents that these statements were not true if they had given a response of “true”.

9 Refers to surgical masks or face coverings. While disposable surgical masks were provided to all SMC distributors. Home-made face coverings were used in some instances.

10 These included local leader, religious leader, health facility staff, community health worker, radio, printed materials (including newspapers, leaflets and banners), television, town announcers, word of mouth (i.e. family or friends), and any other source (including public health campaigns delivered by mobile text messaging).

11 Unweighted proportion.
